# Innovation in early medical education, no bells or whistles required

**DOI:** 10.1186/s12909-020-1947-6

**Published:** 2020-02-07

**Authors:** Cory J. Rohlfsen, Harlan Sayles, Gerald F. Moore, Ted R. Mikuls, James R. O’Dell, Sarah McBrien, Tate Johnson, Zachary D. Fowler, Amy C. Cannella

**Affiliations:** 1 Department of Internal Medicine, University of Nebraska Medical Center (UNMC), 983332 Nebraska Medical Center, Omaha, NE 68198-3332 USA; 2 Department of Biostatistics, College of Public Health, UNMC, Omaha, NE USA; 3 College of Medicine, UNMC, Omaha, NE USA; 4 Department of Rheumatology, UNMC, Omaha, NE USA; 5 College of Allied Health Professions, UNMC, Omaha, NE USA; 6 College of Information Science and Technology, University of Nebraska Omaha, Omaha, NE USA

## Abstract

**Background:**

Despite a paucity of evidence to support a multitude of educational innovations, curricular leaders are pressured to find innovative solutions to better prepare medical students for an evolving twenty-first century health care system. As part of this effort, this study directly compared student-rated effectiveness scores of six different learning modalities.

**Methods:**

Study participants included 286 medical students enrolled in the second-year rheumatology core at a single academic medical center between 2013 and 2017. Students were surveyed at the end of the core with a 15-item questionnaire, and student perceived effectiveness of six different learning modalities were compared.

**Results:**

The modality that outperformed all others was *Live Patient Encounters (LPE)*, with significantly higher student-rated effectiveness scores when compared to the referent modality of Problem-Based Learning (PBL). Using a 5-point Likert scale with responses ranging from “not effective” to “highly effective,” LPE received a mean effectiveness score of 4.77 followed by Augenblick (4.21), PBL (4.11), Gout Racer video game (3.49), Rheumatology Remedy e-module (3.49), and simulation knee injection (3.09).

**Conclusions:**

Technologically advanced novel learning strategies were outperformed in this study by the more traditional active learning modality of LPE. This finding highlights the importance of testing innovative learning strategies at the level of the learner. Three additional conclusions can be drawn from this result. First, conflation of technology with innovation may lead to a myopic view of educational reform. Second, human factors seem to be responsible for the success of LPE and may have far-reaching educational rewards. Third, further applications of LPE should be tested in non-rheumatologic curricula. The relevance of this study is innately tied to the humanities-based application. While a formal qualitative analysis was not performed in this study, preliminary results suggest that live, structured patient interactions in the pre-clinical years of medical education may not only promote the learning of important educational objectives but also foster professional development, empathy, reflection, leadership, agency, and interpersonal skills. This “win-win” scenario (if true) would stand out as a rarity among strategic educational initiatives.

## Background

Integrating novel pedagogical techniques can be challenging to medical educators tasked with curricular reform [1]. While it may be tempting to adopt innovative curricular changes, these efforts are often based on a paucity of evidence. Despite the widespread application of novel educational modalities, direct comparison data are rare, and specific curricular prescriptions are non-existent. Although active learning strategies are considered superior to traditional lecture format, strategic implementation of specific active learning components has been less clearly defined [2]. As a result, curricular leaders are often conflicted with how to integrate small groups, e-learning, and traditional lecture to achieve the best possible learning experience [3, 4]. Unfortunately, evolving modalities of information delivery can merely add to the confusion [5].

Additional barriers to innovation include cultural inertia and limitations in time, finances, faculty, and technological support. Assuming these practical barriers can be overcome, a sense of uncertainty remains due to the inadequacy of comparison data to make relative value decisions with respect to various innovative modalities [6]. Although this should be the most critical factor driving educational innovation, no study to date has tested multiple innovative educational modalities in head-to-head fashion within an already established medical school curriculum.

## Methods

The purpose of this study was to test six active learning modalities and compare learner perceived effectiveness. With the exception of Problem-Based Learning (PBL), traditional lecture had been the mainstay of information delivery in the rheumatology curriculum at the University of Nebraska Medical Center (UNMC) until 2014. From 2014 to 2017, five additional innovative learning strategies were deployed for second year medical students (Table 1).

**Table 1:** Six active learning modalities utilized in the M2 Rheumatology Core at the UNMC College of Medicine. All asynchronous learning modalities were voluntary

Innovative learning modality	Synchronicity of active learning modality	Years in use
1) Live Patient Encounters (LPE)	Synchronous	2014–2017
2) Augenblick cases	Synchronous	2014–2017
3) Problem-Based Learning (PBL)	Synchronous	2000–2017
4) Gout Racer video game	Asynchronous	2014–2017
5) Simulation knee injection	Asynchronous	2014–2015
	Synchronous	2016–2017
6) Rheumatology Remedy e-module	Asynchronous	2016–2017

Our aim was threefold: (1) to implement and test a combination of synchronous and asynchronous active learning components (with purposeful redundancy) in order to enhance the learning experience in the rheumatology curriculum, (2) to integrate these modalities seamlessly, without compromising student satisfaction, and (3) to study how these modalities would be received by students to inform future curricular changes.

Upon completion of the final rheumatology examination, all second year medical students were verbally consented to answer a voluntary and anonymous 15-question survey (embedded in a larger post-core questionnaire) regarding their perceptions of the effectiveness of each learning modality experienced during the core (Appendix 1). This survey was administered yearly at a single academic medical center between 2013 and 2017.

Learning modality effectiveness was assessed using a 5-point Likert scale with responses ranging from “not effective” to “highly effective.” Mean effectiveness scores were then compared between learning modalities using one-way ANOVA with post-hoc pair-wise comparisons and Scheffe’s method to adjust for multiple comparisons. Because PBL had been a staple of active learning within the rheumatology curriculum at UNMC for several years, it was treated as the referent modality in post-hoc comparisons. Statistics were run using Stata SE 14.2 software (Stata Corp, College Station, Texas).

A brief description of each innovative modality is provided below:

1. *Live Patient Encounters (LPE)*

Ten stations were developed including polarized microscopy for crystal analysis, musculoskeletal ultrasound, and eight patients recruited from clinic with representative rheumatic diseases. Patients consented to having their labs, x-rays, and photographs available when appropriate. The class was divided into ten groups and groups rotated through each station in 15-min intervals over a 3 h time period, including a patient break in the middle. During each station, patients were allowed to lead the group through their history, and students were encouraged to ask questions and examine each patient. Faculty facilitators were present to answer medical questions. LPE occurred after each of the representative diseases had been formally taught in lecture. At the end of the encounters, the students completed a low-stakes quiz.

2) *Augenblick*

By definition, augenblick means “blink of an eye” or “moment.” Thirty pathognomonic rheumatic disease pictorial findings (with two associated high yield questions) were presented to students in a Power Point format. Initially, student-led small groups met to work through the augenblick cases. Rheumatology faculty then supervised each student group at a later date to review answers and facilitate discussion.

3) *Problem-Based Learning (PBL):*

Students independently met twice (small groups of 10) to review two electronic cases with timed release of information followed by additional questions. Rheumatology faculty then supervised each student group at a later date to demonstrate how a clinician would work through the cases, answer questions, and highlight the learning objectives. At the completion of the cases, students completed a low-stakes quiz and were given a study guide to review important learning points.

4) *Gout Racer video game:*

Rheumatology faculty teamed with the College of Information Science and Technology at the University of Nebraska Omaha (IST at UNO), to develop a gout-themed video game. Through visually-rich graphics, students were challenged to navigate a dune buggy (the “Gout Racer”) through a series of obstacles and differing terrain representing the pathophysiology, clinical presentation, and treatments of gout. Bonus points and hazards were utilized to visually and audibly reinforce important clinical content. Within each terrain, students had to answer multiple choice questions in order to advance to the next level. Students were given immediate feedback with explanations after each question attempt. This video game was voluntary and no formal grade or assessment was linked to student performance. As an incentive to play the game, students were informed that questions from Gout Racer would appear on the final exam.

5) *Simulation Knee Injection:*

Students were asked to view a preparatory video on joint injection techniques followed by dedicated time in the simulation lab to practice knee injections. No faculty supervision was assigned during the first 2 years of the study. In response to student feedback, this activity was modified in the latter 2 years of the study to include a 20-min lecture with faculty demonstration of injection technique followed by direct faculty supervision of simulated knee injections.

6) *Rheumatology Remedy e-module:*

The e-learning lab at the IST at UNO supported the development of an interactive, inter-professional, e-learning module that was accessible to students throughout the entirety of the rheumatology core. The module highlighted both pharmacologic and non-pharmacologic therapeutics. This formative assessment included 100 multiple choice questions with immediate feedback. The module was voluntary, and no formal grade or assessment was linked to student performance.

## Results

From 2014 to 2017 there were 286 student survey respondents with a total response rate of 57.4%. Individual response rates by year were as follows: 2014 (61.8%), 2015 (89.1%), 2016 (48.4%), 2017 (29.6%).

### Effectiveness of learning modality

**Fig. 1:**
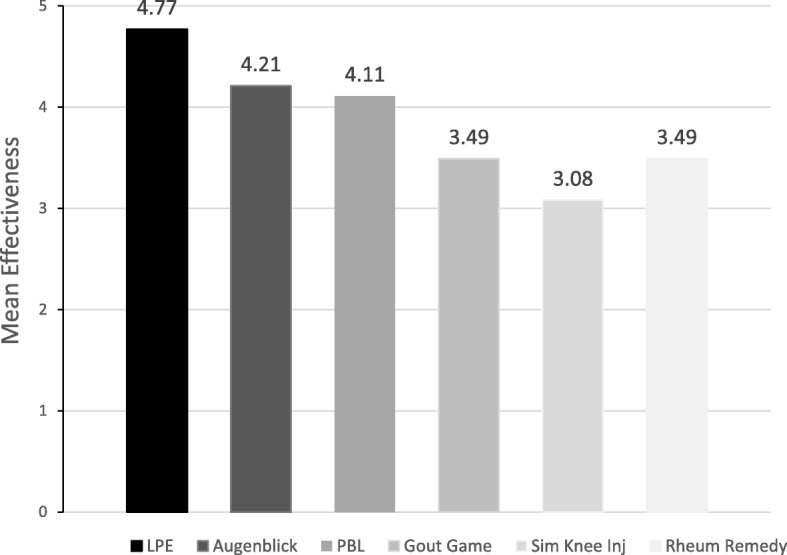
Student perceived effectiveness of individual learning modalities based on survey data from 2014 to 2017

Of the six modalities tested, LPE was associated with the highest student perceived effectiveness (Fig. 1) with a mean effectiveness score of 4.77 followed by Augenblick (4.21) and PBL (4.11). The three least effective innovative modalities were the Gout Racer video game (3.49), the Rheumatology Remedy e-module (3.49), and the simulation knee injection (3.09). Mean effectiveness scores with standard deviations are summarized in Table 2.

**Table 2:** Summary of mean effectiveness scores as reported by students. Mean effectiveness scores were analyzed with one-way ANOVA using post-hoc adjustments for pair-wise comparisons relative to PBL (“baseline”) and included Scheffe’s method for multiple comparison adjustment

Innovative learning modality	Modality effectiveness		
	Mean effectiveness score (+/− SD)	*P*-value (vs. PBL)	% of students reporting modality to be at least moderately effective (% highly effective)
Live Patient Encounters (LPE)	4.77 (+/− 0.55) *n* = 286	< 0.001	96.9% (81.5%)
Augenblick	4.21 (+/− 0.92) n = 286	0.901	84.3% (45.5%)
Problem-Based Learning (PBL)	4.11 (+/− 1.01) *n* = 285	Referent	80.7% (43.2%)
Gout Racer video game	3.49 (+/− 1.10) *n* = 275	< 0.001	52.7% (17.1%)
Simulation Knee Injection	3.09 (+/− 1.10) *n* = 276	< 0.001	36.6% (8.7%)
Rheumatology Remedy e-module	3.49 (+/− 1.14) *n* = 94	< 0.001	51.0% (22.3%)

Based on analysis with ANOVA pair-wise comparisons, LPE outperformed PBL in student-rated effectiveness (*p* < 0.001). While Augenblick had a higher mean effectiveness score, the difference in relation to PBL was not statistically significant.

Of note, 97% percent of students perceived LPE to be highly or moderately effective and LPE was the only modality perceived by a majority of students to be highly effective (Table 2). A qualitative analysis of survey responses was not performed in this study. Examples of student responses can be found in Appendix 2.

## Discussion

As the needs of the twenty-first century health care system continue to evolve, medical students must be trained to meet a multitude of professional demands. Given restraints on time and resources, curricular reform leaders have placed an emphasis on innovation and novel learning strategies [7]. As part of this national effort, UNMC has implemented five innovative learning modalities within the rheumatology core and tested them against PBL- a benchmark of active learning within the prior UNMC curriculum.

Although effectiveness scores varied amongst each of the educational modalities, one clearly stood out from the rest. LPE consistently outperformed the other active learning strategies over the course of 4 years and was well received by greater than 97% of students. The degree to which this occurred is somewhat surprising as most of the active learning literature to date emphasizes the importance of self-directed study, inquiry-based learning (e.g. PBL), blended learning (with use of asynchronous e-learning), simulation, and gamification [8–12]. LPE is not well represented in the literature, has no technological prowess, and only requires 3 h of dedicated student time; yet it was able to outperform the more widely accepted, technologically-advanced, asynchronous, and traditional active learning strategies (including PBL). This irony highlights the importance of testing innovative learning modalities at the level of the learner.

Interestingly, the utility of LPE has been well documented in rheumatologic curricula for decades but its scope of use pales in comparison to other innovative learning modalities with less supporting evidence but greater technological appeal [13, 14]. In an era where technology is often conflated with innovation and evidence is in relatively short supply, curricular leaders are left to “innovate” based on cultural readiness and limited resources. As such, innovations may be adopted more so on the basis of popular trends and consensus rather than merit. Moreover, modalities requiring significant investment in time and energy (like LPE) are unlikely to gain traction without substantial evidentiary support. Ultimately it seems “proof of concept” is not good enough for curricular prescriptions, and head-to-head comparison data is required to make relative value decisions. To our knowledge, we are the first to study LPE in this manner.

Our results may highlight a potential bias within educational reform initiatives that favors technology. Ultimately, technological advances may be a step away from humanism and could compromise the learning of important cross-cutting domains [15] such as professional development, empathy, and agency within the health system sciences. This perspective is important in balancing an otherwise myopic view of educational reform.

It should not come as a surprise that medical students crave patient contact particularly in their pre-clinical years. Although we hesitate to make claims attributing the entirety of LPE’s success to the human factors element, the association is difficult to ignore as this is the main difference between LPE and other innovative educational modalities. Live patients activate the affective domain of learning [16] in ways that other modalities simply cannot. This domain is important in assimilating long-term retention of knowledge and facilitating student identity formation and professional development [17]. The hidden curriculum is now widely recognized as an important factor in undergraduate medical education, and early exposure to real patients is strongly advised as one strategy to accomplish this aim [18]. As such, we suggest that LPE may not only promote the learning of educational objectives but also foster professional development, empathy, reflection, leadership, agency, and interpersonal skills [19–21]. This “win-win” scenario would stand out as a rarity among strategic educational initiatives [22].

More research is needed to investigate long term outcomes of LPE, and replication of our findings at external sites is recommended. Additionally, we recommend “proof of concept” trials in non-rheumatologic curricula as the application of this modality may not be generalizable to the learning of representative diseases outside of rheumatology.

Directly testing the acquisition of medical knowledge with LPE would also be an important metric to consider. Assessment of knowledge acquisition was not feasible in this study primarily because the curriculum at UNMC has a competing priority of planned redundancy that precludes attribution of knowledge to any singular modality. For instance, gout was intentionally taught in LPE, PBL, Augenblick, Rheumatology Remedy, and the Gout Racer video game. Any attempt to isolate the effects of one modality would defeat the purpose of having multiple exposures to the same educational content. It should be noted, however, that learner satisfaction has been indirectly linked to knowledge acquisition when evaluating novel educational modalities [23]. Thus, when knowledge cannot be directly assessed, student-rated effectiveness may be an appropriate surrogate metric.

Another limitation of this study is that it may be subject to survey sampling bias. With greater than a 50% response rate, we feel respondents accurately represented students at UNMC although we do not have demographic data to differentiate respondents from non-respondents. More importantly, students at UNMC may not be representative of all medical students. Similarly, the individual modalities described may not reflect the practices or implementation strategies used at other institutions.

Despite these limitations, we feel the rewards of implementing LPE into rheumatologic curricula outweigh the costs in time, management, and organization (Appendix 3). This innovative modality was well received by students within our institution, and future rewards of LPE may be yet to be seen. As we continue to navigate an evolving health system, educators should strive for innovative learning solutions that foster knowledge acquisition, professional identity formation, and learner satisfaction.

## Data Availability

All data generated or analyzed during this study are included in this published article. Supplementary data sets can be found in Appendix 4 and Appendix 5.

## Appendix 1

### Survey from 2017 with some parts excluded

1. Was the simulation experience (Arthrocentesis) in the lecture hall an effective way to learn the material?
  i. Not effective
  ii. Slightly effective
  iii. Neutral
  iv. Moderately effective
  v. Highly effective
2. Did you play Gout Racer? (Yes, No)
3. How many times did you play Gout Racer? (0, 1, 2, 3, 4, 5+)
4. Was Gout Racer an effective way to learn the material?
  i. Not effective
  ii. Slightly effective
  iii. Neutral
  iv. Moderately effective
  v. Highly effective
5. Do you have any suggestions for improvement for Gout Racer?
6. Did you play Rheumatology Remedy? (Yes, No)
7. How many times did you play Rheumatology Remedy? (0, 1, 2, 3, 4, 5+)
8. Do you have any suggestions for improvement for Rheumatology Remedy?
9. Was Rheumatology Remedy an effective way to learn the material?
  i. Not effective
  ii. Slightly effective
  iii. Neutral
  iv. Moderately effective
  v. Highly effective
10. Were the week one small group cases an effective way to learn the material?
  i. Not effective
  ii. Slightly effective
  iii. Neutral
  iv. Moderately effective
  v. Highly effective
11. Were the Augenblick cases an effective way to learn the material?
  i. Not effective
  ii. Slightly effective
  iii. Neutral
  iv. Moderately effective
  v. Highly effective
12. Please comment on what you liked or disliked about the week one small group mini-cases and Augenblick.
13. Were the week two small group cases (patient stations) an effective way to learn the material?
  i. Not effective
  ii. Slightly effective
  iii. Neutral
  iv. Moderately effective
  v. Highly effective
14. Please comment on what you liked or disliked about the week two small group mini-cases (patient stations).
15. Are there any other suggestions for improving your learning experience?

## Appendix 2

### Selected student survey responses regarding Live Patient Encounters

- “Best thing ever. I could remember the diseases because I could put a face to the disease, and thus the findings. It was inspiring to hear the real patient stories.” (M2 from 2014)
- “This was one of the most effective forms of education I have been presented while at UNMC. What better way is there to learn medicine than to see real patients with real diseases?” (M2 from 2014)
- “Talking with patients who suffered from the diseases was by far the best small group experience we have ever had in medical school. I almost categorically do not enjoy small group, but this was really worth our time. These patients will probably remain in my memory for the rest of my career.” (M2 from 2014)
- “I will never forget these cases. I also learned how to empathize with patients in a way that I never would learn in a classroom.” (M2 from 2014)
- “Patient small groups were the best thing we have done in medical school.” (M2 from 2014)
- “I LOVED the day the patients came. It brought back the humanity of medical school, and I will always remember those patients.” (M2 from 2014)
- “The real patient interaction was phenomenal, and permanently impacted the way I will view rheumatology cases for the rest of my career.” (M2 from 2014)
- “I am very grateful for these patients being willing to share their stories with so many students/strangers. It made the material much more tangible, and was a great opportunity to put a real face on some very difficult diseases. It’s easy for us to just memorize the facts and completely miss the human factor in these diseases. Awesome part of this core!” (M2 from 2014)
- “As I was taking the test, I could picture the specific patients as I was thinking about the disease. SO BENEFICIAL!” (M2 from 2014)
- “One of the best forms of medical education that I have experienced in the first 2 years. Wish we had this for every core. Keeps our head focused in the right direction. Gives us a chance to put a face & experience to a disease. Very very very helpful and highly recommended to continue (and advise other faculty to do something similar for their cores if possible).” (M2 from 2015)
- “So far, this was the highlight of my M2 year. It was extremely helpful and I can tell I will remember the things we discussed with the actual patients for a long time. Even during the exam, if there was a lupus question, I would envision the patient with lupus and some of the things that she talked about, and did the same for scleroderma, dermatomyositis, etc.” (M2 from 2015)
- “The patient stations were awesome and this was the most effective way of learning for me. There are some things that are definitely in my long term memory. I wish we could do this for all of the cores! I also loved adding the human part of medicine back into what we were learning.” (M2 from 2015)
- “I WISH ALL CORES HAD THIS SET-UP OF PATIENT INTERACTION!!! Sorry for the all-caps but I learned so much more in these three hours of patient interaction than I would have just starting at my lecture notes. For the benefit of student learning, continue to do this next year, and please tell other core directors how helpful this patient interaction was for visual and tactile learners. I do not have one of those “see it once” memories, so this experience was invaluable, and I believe it will stay in my memory for boards, 3rd year rotations, and the duration of my medical career.” (M2 from 2015)
- “It would have been easy to get the impression that rheumatology (has a) bunch of obscure diseases with a bunch of textbook pictures to memorize, but getting to interact with people living with these conditions helped make the importance of this topic real. Getting to see and feel how rheumatologic diseases change a person’s body and then hearing from the patients about how it has affected their lives was a memorable and valuable educational experience.” (M2 from 2016)
- “Meeting the patients made the material we were learning about real and applicable. They made me want to study harder. I can remember the information much better now and am so thankful for the patients who came and shared their stories!” (M2 from 2016)
- “Seeing patients with each disorder really solidified my understanding of concepts from lecture. It was also a nice break from the grind of studying and listening to lectures, and a good reminder of what we are working for.” (M2 from 2016)
- “This was the highest yield clinical/classroom overlap thus far in medical school.” (M2 from 2016)
- “This was a VERY effective way of learning by being able to hear from patients’ own voices what it’s like to live with these different disease. This was one of the most valuable learning experiences we’ve had in the first two years.” (M2 from 2017)
- “The patient stations were excellent all around. Nothing beats seeing a condition/disease first hand. I feel very grateful to the patients who volunteered for that experience.” (M2 from 2017)
- “I loved the experience.” (M2 from 2017)

## Appendix 3

### Core Director Considerations

Recommended timeline and steps required for the successful implementation of Live Patient Encounters (LPE) into a medical school curriculum

1. Identify patients with representative diseases from clinics (4 months prior to encounter)
2. Invite patients to participate via telephone, with immediate email confirmation (4 months prior to encounter)
3. Prepare power-point with images from patient records for student review prior to encounter
4. Prepare quiz for students upon completion of encounter
5. Contact patients to confirm participation and give logistics for the day (2 weeks prior to encounter)
6. Day of encounter
  - Administrative assistants and nursing facilitate getting patients from designated meeting point to encounter location
  - Faculty transport ultrasound equipment and supplies and microscope (with MSU/CPPD slides) to encounter location
  - Faculty and administrative assistants monitor time and alert room changes
  - Refreshments are provided to patients and faculty (rolls, coffee, tea)
7. Students sign a thank you card for each patient
8. Send thank you letter from Block Director and Dean with selected student comments, students’ signed card and $50 reimbursement (1 month after encounter)

Author contact information: Please direct any specific questions to Amy C. Cannella, MD, MS at acannella@unmc.edu

A summary of administrative, faculty, technological, and financial requirements estimated for each innovative learning modality can be found in Table 3 in Appendix 4.

## Appendix 4

**Table 3:** Innovative educational modality effectiveness scores 2014–2017 (Fig. 1)

	2014	2015	2016	2017	sum	% sum	Mean Effectiveness Score
Live patient							
Unanswered	0	0	0	0	0	0	
Not Effective	0	0	1	0	1	0.34965035	
Slightly Effective	0	0	2	0	2	0.6993007	
Neutral	2	2	2	0	6	2.0979021	
Moderately Effective	5	23	7	9	44	15.3846154	
Highly Effective	69	89	47	28	233	81.4685315	
					286		4.769230769
PBL							
Unanswered	0	1	0	0	1	0.35087719	
Not Effective	3	1	0	0	4	1.40350877	
Slightly Effective	6	13	8	1	28	9.8245614	
Neutral	7	13	2	1	23	8.07017544	
Moderately Effective	33	36	21	17	107	37.5438596	
Highly Effective	27	50	28	18	123	43.1578947	
					285		4.112280702
Gout Game							
Unanswered	4	5	0	2	11	4	
Not Effective	0	4	1	1	6	2.18181818	
Slightly Effective	16	13	8	7	44	16	
Neutral	13	38	13	16	80	29.0909091	
Moderately Effective	29	35	24	10	98	35.6363636	
Highly Effective	14	19	13	1	47	17.0909091	
					275		3.494545455
Augenblick							
Unanswered	0	0	0	0	0	0	
Not Effective	1	2	0	1	4	1.3986014	
Slightly Effective	1	9	5	1	16	5.59440559	
Neutral	4	14	6	1	25	8.74125874	
Moderately Effective	28	47	21	15	111	38.8111888	
Highly Effective	42	42	27	19	130	45.4545455	
					286		4.213286713
Simulation							
Unanswered	6	4	0	0	10	3.62318841	
Not Effective	6	19	2	4	31	11.2318841	
Slightly Effective	9	16	7	7	39	14.1304348	
Neutral	26	35	27	17	105	38.0434783	
Moderately Effective	20	33	19	5	77	27.8985507	
Highly Effective	9	7	4	4	24	8.69565217	
					276		3.086956522
Rheum Remedy							
Unanswered	N/A	N/A	1	1	2	2.12765957	
Not Effective	N/A	N/A	3	2	5	5.31914894	
Slightly Effective	N/A	N/A	11	2	13	13.8297872	
Neutral	N/A	N/A	13	15	28	29.787234	
Moderately Effective	N/A	N/A	17	10	27	28.7234043	
Highly Effective	N/A	N/A	14	7	21	22.3404255	
					94		3.489361702
Figure 1 Percentages (not pictured)	Not Effective	Slightly Effective	Neutral	Moderately Effective	Highly Effective		
Live Patient Encounters	0.35	0.7	2.1	15.38	81.47		
Augenblick	1.4	5.59	8.74	38.81	45.45		
PBL	1.4	9.82	8.07	37.54	43.16		
Gout Game	2.18	16	29.09	35.64	17.09		
Sim Knee Inj	11.23	14.13	38.04	27.9	8.7		
Rheum Remedy	5.32	13.83	29.79	28.72	22.34		
Figure 1 Means (pictured)	Mean Effectiveness Score						
Live Patient Encounters	4.77						
Augenblick	4.21						
PBL	4.11						
Gout Game	3.49						
Sim Knee Inj	3.08						
Rheum Remedy	3.49						

## Appendix 5

**Table 4:** Summary of administrative, faculty, technological, and financial requirements estimated for each innovative learning modality

Modality	Dedicated # hours within the curriculum	Time commitment prior to implementation	Financial commitment prior to implementation^a^	Time commitment after implementation	Financial commitment after implementation^a^	# faculty required for the day	Additional considerations that may affect time and/or cost of learning modality
Live Patient Encounters	3	6 h (planning/development) 2 h (recruitment) 3 h (administration)	$ 0	6 h (planning/development) 2 h (recruitment) 3 h (administration)	$ 500	10	Musculoskeletal ultrasound Polarized Microscope Patient consents and liability Patient thank you letters Patient reimbursement
Augenblick	1	5 h (planning/development) 1 h (administration)	$ 0	1 h (faculty) 1 h (administration)	$ 0	12	Requires strategic development for purposeful redundancy Consider integration with PBL small groups
PBL	5	15 h (planning/development) 2 h (administration)	$ 0	2.5 h (faculty) 2 h (administration)	$ 0	12	Requires strategic development for purposeful redundancy Consider integration with Augenblick small groups
Gout Racer Game	1	120 h (planning/development)	$ 5000	1 h (faculty)	$ 500	1	Requires initial and ongoing technological support^a^
Simulation Knee Injection	1	4 h (planning/development) 1 h (administration)	$ 2400 (knee simulators)	0.5 h (faculty)	$ 10 (supplies)	5	Storage of simulator knees
Rheum Remedy E-module	1	120 h (planning/development)	$ 5000	1 h (faculty)	$500	1	Requires initial and ongoing technological support^a^

^a^These are costs incurred in addition to faculty and administrative support for the time required for development, implementation, and ongoing administration of each educational modality

## Abbreviations

LPE: Live Patient Encounter
PBL: Problem-Based Learning
UNMC: University of Nebraska Medical Center

## Glossary

Active learning strategy: An approach to instruction in which students directly engage in the learning process
Live Patient Encounters (LPE): An active learning strategy facilitated in a non-clinical environment for pre-clinical students based on structured interactions with real patients with representative diseases (not actors)
Augenblick: An active learning strategy involving small groups of students who work through high yield, pathognomonic findings of medical diseases
Problem-Based Learning (PBL): An active learning strategy in which students learn about a subject in small groups as they investigate and solve open-ended, clinical problems
Blended learning: A style of hybrid education in which students learn via a mixture of electronic and online media as well as traditional face-to-face instruction
Curricular prescription: A best practice recommendation for instructional methodology that is unique and specific to the learner, content, and venue of education
